# Associated factors study into the belated screening for leprosy in Benin

**DOI:** 10.1371/journal.pntd.0010533

**Published:** 2022-06-23

**Authors:** Ronald Sètondji Gnimavo, Ghislain Emmanuel Sopoh, Parfait Djossou, Esaï Gimatal Anagonou, Gilbert Adjimon Ayélo, Anita Carolle Akpéédjé Wadagni, Yves Thierry Barogui, Jean Gabin Houezo, Roch Christian Johnson

**Affiliations:** 1 Public Health Regional Institute- Comlan Alfred Quenum, Ouidah, University of Abomey-Calavi, Benin; 2 Center for Leprosy and Buruli’s Ulcer Screening and Treatment « Raoul et Madeleine Follereau » of Pobè, Benin; 3 National Leprosy and Buruli’s Ulcer Control Program, Health Department, Benin; 4 Interfaculty Training Center for Environmental Research for Sustainable Development, University of Abomey-Calavi, Benin; 5 Center for Buruli’s Ulcer Screening and Treatment « Le Luxembourg » of Allada, Benin; 6 Center for Buruli’s Ulcer Screening and Treatment of Lalo, Benin; 7 Fondation Raoul Follereau, Health Department, Paris, France; Federal University of Sergipe: Universidade Federal de Sergipe, BRAZIL

## Abstract

**Introduction:**

In the absence of early treatment, leprosy, a neglected tropical disease, due to *Mycobacterium leprae or Hansen Bacillus*, causes irreversible grade 2 disability (G2D) numerous factors related to the individual, the community and the health care system are believed to be responsible for its late detection and management. This study aims to investigate the factors associated with belated screening for leprosy in Benin.

**Methods:**

This was a cross-sectional, descriptive, and analytical study conducted from January 1 to June 31, 2019, involving all patients and staff in leprosy treatment centers and public peripheral level health structures in Benin. The dependent variable of the study was the presence or not of G2D, reflecting late or early screening. We used a logistic regression model, at the 5% threshold, to find the factors associated with late leprosy screening. The fit of the final model was assessed with the Hosmer-Lemeshow test.

**Results:**

A number of 254 leprosy patients were included with a mean age of 48.24 ± 18.37 years. There was a male dominance with a sex ratio of 1.23 (140/114). The proportion of cases with G2D was 58.27%. Associated factors with its belated screening in Benin were (OR; 95%CI; p) the fear of stigma related to leprosy (8.11; 3.3–19.94; <0.001), multiple visits to traditional healers (5.20; 2.73–9.89; <0.001) and multiple visits to hospital practitioners (3.82; 2.01–7.27; <0.001). The unawareness of leprosy by 82.69% of the health workers so as the permanent decrease in material and financial resources allocated to leprosy control were identified as factors in link with the health system that helps explain this late detection.

**Conclusion:**

This study shows the need to implement strategies in the control programs to strengthen the diagnostic abilities of health workers, to improve the level of knowledge of the population on the early signs and symptoms of leprosy, to reduce stigmatization and to ban all forms of discrimination against leprosy patients.

## Author summary

Leprosy is one of the neglected tropical diseases that manifests itself by a cutaneous and neurological attack whose evolution in the absence of an early detection and management is inevitably towards an acquired handicap. This handicap is responsible for significant physical, psychological and socio-economic suffering which alters the quality of life of the patient.

In Benin, the epidemiological profile of leprosy is marked by a high and increasing proportion of new cases of leprosy with grade 2 disability and a high proportion of multibacillary leprosy cases. These indicators are markers of late detection of leprosy. In view of the new objectives of the new roadmap developed by WHO for the period 2021–2030, leprosy control remains a major health challenge for the actors of the epidemiological surveillance system. It is for this reason that we undertook this study to better understand the factors responsible for the late detection of leprosy in our country in order to propose relevant and innovative strategies and interventions to achieve the objectives of the WHO roadmap and also to participate in the improvement of the quality of life of leprosy patients.

Our approach is motivated by the ambition to improve the health status of people affected by neglected tropical diseases, in this case leprosy.

## Introduction

Leprosy is a chronic infectious disease caused by *Mycobacterium leprae* **[1].** It primarily affects the skin, respiratory tract epithelium, and peripheral nerve tissues, and then incurs visible physical deformities known as "grade 2 disabilities" (G2D), if not treated early **[2–3]**. It is estimated that more than 3 million people worldwide live with disabilities due to leprosy **[4]**, with 11323 new cases being reported in 2018 **[5]**. Such G2D are direct markers of belated screening for new leprosy cases. They are indicative of the low level of community awareness of the early signs and symptoms of leprosy and reflect the inability of health systems to recognize and treat leprosy at an early stage of the disease **[6–7]**. From an epidemiological point of view, this means that the patient has remained undiagnosed for a long time and has therefore had ample time to develop irreversible complications. These complications cause significant physical, psychological and socio-economic consequences that may impair the quality of life of the victim and expose affected persons to begging **[8–10]**. The severity of the clinical damage done by leprosy and its numerous impacts on the lives of people affected by leprosy constitute a major health challenge for projects, programs and organizations involved in leprosy control. In response to this, the World Health Organization developed a new roadmap for neglected tropical diseases in April 2019, with a key goal of reducing the number of new leprosy cases detected with G2D to below one case per 1 million population by 2030 **[11]**. The epidemiological pattern of leprosy in Benin is marked by a high prevalence of new leprosy cases with G2D at screening. The proportions reported by the National Leprosy and Buruli’s Ulcer Control Program (NLBUCP) of Benin have been increasing since 2015 and are well above the key targets set by the WHO roadmap for 2030. The percentage of G2D cases among new cases ranged from 18.13% to 32% between 2015 and 2018 **[12]**. We posit that solving this health problem in Benin, requires a better understanding of the set of factors that are at the core of the high prevalence of G2D in Benin; hence the present study, whose objective was to identify the factors associated with late detection of leprosy in Benin.

### Framework and study methods

#### Ethics statement

The survey was carried out after authorization for data collection was granted by the National Health Research Ethics Committee (Reference n°21/MS/DC/SGM/DRFMT/CNERS/SA).

In the site, the objectives of the survey were clearly explained to the participants and the questionnaires were administered only after having collected their free and informed consent. Informed consent was obtained orally from all participants.

We also guaranteed the confidentiality of the information provided to us, which will only be used for the purposes of this study and will remain anonymous. We confirm that all methods used in this observational study complies with all research guidelines and regulations. All the experiment protocol for involving humans in this manuscript are in accordance to guidelines of national/international/institutional and the Declaration of Helsinki. With regards to childrens to be enroll in this study, it was planned to obtain their parents and child’s guardians’ free consent. However no child were found during the survey.

### Study setting

The study was held in Benin in leprosy treatment centers (LTCs) and peripheral health structures run by specialized health workers called Leprosy Nurse Supervisors (LNS).

**Fig 1:**
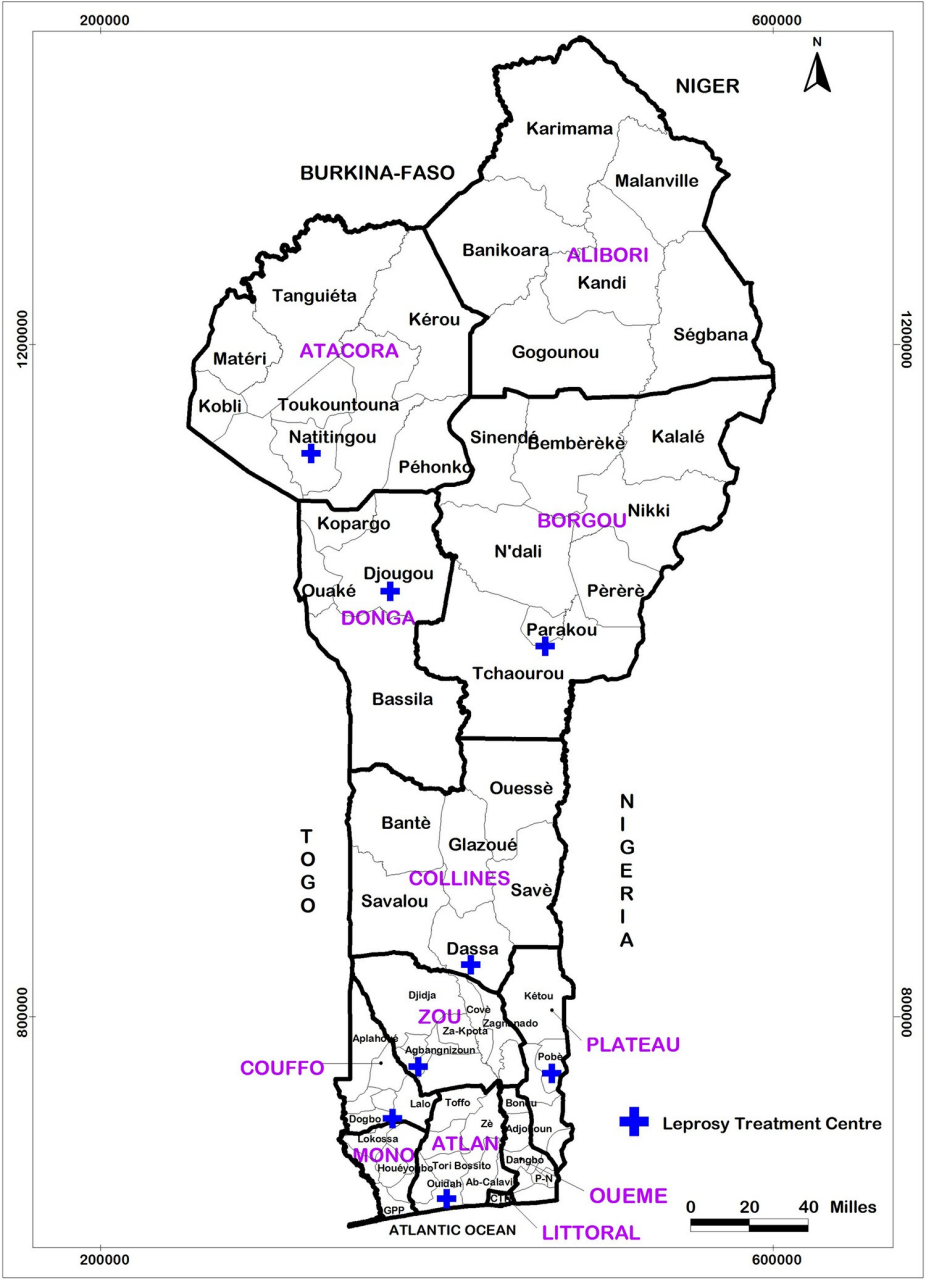
Administrative map of Bénin, showing the location of the leprosy treatment centres in the departments. The base layer of the map used in [Fig 1] was obtained from DIVA-GIS (https://www.diva-gis.org/gdata).

There are eight LTCs, which are considered to be peripheral level health structures, under the technical supervision of the National Leprosy and Buruli Ulcer Control Programme (NLBUCP). The LTCs are located throughout the country, notably in Pobè, Ouidah, Madjrè, Davougon, Dassa-Zoumé, Parakou, Djougou and Natitingou (Fig 1).

These different centers work in collaboration with the district health centers, which they supervise.

LTCs are specialized in the management, validation of new cases (screening, diagnosis) and management (follow-up of outpatient treatment, hospital management of severe cases, psycho-social follow-up and community rehabilitation) of leprosy.

The LNS are qualified agents, trained by the NLBUCP for the screening and management of leprosy. Each municipality has an LNS. The 77 LNS work in collaboration with the LTCs and other health structures at the peripheral level. The NLBUCP of Benin ensures the development of policies and strategies, the mobilization of resources for the implementation of activities, monitoring, evaluation and the organization of research activities. It organizes regular formative supervision (every six months) of the LTCs and LNS to validate detected leprosy cases and to strengthen the skills of all actors involved in the epidemiological surveillance of leprosy. It also monitors and assesses the variable leprosy control activities.

### Type and study period

This was a cross-sectional, analytic study that took place from January 1, 2019, to June 31, 2019.

### Study population

The target population consisted of all people affected by leprosy (PAL) screened and treated in the various LTCs in the Republic of Benin from 2017 to 2018.

The secondary targets were made up of:

- Health workers involved in care activities at the public peripheral level health structures
- Health workers in the LTCs
- LNS
- People living in leprosy patient communities
- The Coordinator of the NLBUCP.

### Selection criteria

All clinically confirmed leprosy cases validated by the National Leprosy and Buruli Ulcer Control Program during training supervision of LTCs and LNSs involved in the epidemiological surveillance of leprosy in Benin

All PALs in Benin who gave free and informed consent to the investigators to participate in the study were counted.

Not included were those who had declined to participate in the survey

### Sampling

#### Sampling methods and techniques

The non-probability method with the convenience sampling technique was used for the selection of PALs meeting the selection criteria.

For the selection of secondary targets:

- Health centers were selected using random sampling. For operational reasons related to the limited availability of resources, we randomly selected two health centers in each department.
- In each of the centers thus selected, we chose the health workers who had participated in the study for convenience.
- In the LCTs, the managers were included by reasoned choice and the nurses and support staff by convenience.
- LNS in the departmental capitals and most accessible municipalities, as well as the NLBUCP coordinator, were included by reasoned choice.
- Individuals living in PAL communities were identified by convenience. Thus, we entered the first houses in the vicinity of the residence of the first five leprosy patients found in each department and interviewed the first consenting adult encountered in each house.

### Sample size

#### Primary targets

Were tracked 254 cases in the field out of the 296 (146 in 2017 and 150 in 2018) validated by the NLBUCP over the past two years.

### Secondary targets

- Health workers: in each health center, we surveyed two health workers (a total of 48) into of the twelve departments of Benin.
- LTCs health workers: four workers per LTC were involved, for a total of 32 workers
- Leprosy Nurse Supervisors: two LNS were identified per department, for a total of 24.
- Community members: we administered our questionnaire to five individuals in each department, for a total of 60 people living in the immediate environment of leprosy patients.
- NLBUCP coordinator: he is alone

### Data sources

They were provided by the:

- Leprosy records or "Tomes Sommiers" available in the LTCs.
- Records of people affected by leprosy

### Variables

The dependent variable in our study was late screening, determined by whether or not the person with leprosy had G2D at diagnosis. It was coded as 1 = "Yes" in patients with G2D at screening and 0 = "No" in those without G2D or with disability grade less than 2.

The independent variables consisted of three different groups of factors related to the leprosy patient, the health system, and the community, respectively.

### Factors related to the people affected by leprosy

#### Socio-demographic and economic factors

age, sex, main occupation of the respondents or profession, level of education, ethnicity and religion, mode of screening and socio-economic status of the leprosy patient. This last parameter was determined using the Principal Component Analysis (PCA) method described by Filmer and al **[13]**. From the assets owned and the household attributes we calculated a score which was then divided into tertiles. The first tertile corresponded to the "poor", the second to the "average" and the third to the "rich".

#### Factors related to people affected by leprosy knowledge and perceptions

these include awareness of the early signs and symptoms of leprosy, knowledge of the existence of the LTCs, the distance between the health center and the house, the cost of consultation in the LTCs, the patient’s perception of the cause of leprosy at the onset of the signs and symptoms of the disease (natural or metaphysical cause, i.e., a spell or divine punishment).

#### Factors related to the therapeutic pathway of the leprosy patient

these include the first recourse at the onset of clinical signs of the disease (self-medication, health workers or traditional healers), the time elapsed between the onset of clinical signs of leprosy and the first medical consultation, the time elapsed between the onset of clinical signs and the diagnostic confirmation of leprosy at the LTCs or by health workers, the number of traditional healers, and the number of health workers seen before the diagnostic confirmation.

### Factors related to the health system

#### Factors related to the health workers in the health structures

age, sex, type of health worker, years in the field, and module on leprosy in training schools, in-service training on leprosy, knowledge of the signs and symptoms of leprosy, knowledge of the LTCs in Benin, attitude adopted when consulting a patient with spots, sores and neural disorders.

#### Factors related to the NLBUCP

this concerns the operability (availability and organization of resources) and functionality (standards, processes, results) of the NLBUCP.

### Community factors

#### Factors related to the individuals’ knowledge of leprosy in the community

this includes knowledge of the signs and symptoms, the causative agent, mode of transmission of leprosy, knowledge of the existence of LTCs, and the individual’s participation in a leprosy awareness session.

#### Factors related to the existence of stigma in the community

These take into account the attitudes and practices of individuals towards leprosy patients. This includes whether or not they greet a leprosy patient, they sit around the same table to eat with such patient and whether or not they marry a leprosy patient.

### Data collection

#### Techniques and tools

We conducted a questionnaire survey to collect the data for our study from PAL, health workers from the different health structures. We used a questionnaire and a structured interview guide that were drafted after a review of the relevant literature, to gather not only sociodemographic data, information on the knowledge, attitudes and practices of community members towards those affected by leprosy, but also information on aspects related to the factors associated with the Benin National Leprosy and Buruli Ulcer Control Program as described above (operationality and functionality). The interviews were led by two interviewers and one participant. In addition a direct interview were done with the LNS using a guide.

For data related to the NLBUCP National Coordinator, we also proceed by a direct interview

Interviewers were all trained and linguistically competent in the local dialects. All interviews were audiotaped, translated and transcribed into Word before being analyzed.

All of the interviewers involved in this study were trained and the tools used were pre-tested before the actual collection began.

### Treatment and data analysis

After data collection, the forms were manually sorted to verify the completeness and consistency of the data collected. The data were entered using Epi Info software version 7.2.0.1 and analyzed with Stata 11 software. The location map of the LTCs was made using ArcView GIS 3.2 software. For each LTCs, the geographical details were taken and projected in the background map that we had downloaded for free from the DIVA GIS website (https://www.diva-gis.org/).

The variables were described in terms of the characteristics of leprosy patients and health center workers in the peripheral health structures. Proportions were given for the qualitative variables. Quantitative variables were expressed as means followed by standard deviation for those with a normal distribution. The medians and extremes (minimum and maximum) were determined for quantitative variables with a non-symmetric distribution.

The qualitative responses recorded during the in-depth interviews were translated in full and transcribed into French by two interviewers. In the event of disagreement between the two interviewers, the original transcripts and recordings were reviewed until a consensus was reached.

### Analytical aspect

Univariate analysis consisted of comparisons of proportions between late screening and independent variables with raw Odds Ratios (OR) and their 95% confidence intervals (95% CI). Interactions and confounding factors were searched for. The threshold of significance is 5%.

### Multivariate analysis

In multivariate analysis, we used a top-down stepwise logistic regression model to identify factors associated with late leprosy screening. Variables with a significance level below 20% in the univariate analysis were entered into the initial logistic regression model. Variables with a significance level of less than 5% were included in the final model. The fitness of the final model was assessed with the Hosmer-Lemeshow test.

## Results

### Sociodemographic and economical features

A total of 254 PALs were included in the study. The mean age of the PALs was 48.24 ± 18.57 years. The sex ratio was 1.23 (140/114) in favor of men. The affected persons were uneducated (illiterate) in 83.07% (211/254) of cases. In 59.06% (150/254), patients included in the current study had a grade 2 disability at screening, i.e., they were screened late. Most of them were farmers (147/254, i.e. 57.87%) and lived in rural areas (242/257, i.e. 95.28%) (Table 1).

**Table 1:** Univariate analysis of the link between late screening for leprosy and sociodemographic, economic and clinical features of leprosy-affected persons in Benin from 2017 to 2018.

Variables	Headcount (n = 254)	Frequency (%)	Odds Ratio	[CI _95%_ OR]	p value
**Age**					
≤ 35 years	70	27.56	1	-	-
˃ 35 years	184	72.44	2.30	[1.31–4.033]	**0.004**
**Sex**					
Female	114	44.58	1	-	-
Male	140	55.12	1.24	[0.75–2.057]	0.394
**Educational level**					
Educated	43	16.93	1	-	**-**
Uneducated	211	83.07	2.32	[1.19–4.52]	**0.013**
**Profession**					
Housekeeper	33	12.99	1	-	**-**
Farmer	147	57.87	0.71	[0.31–1.59]	0.403
Retailer	29	11.42	0.40	[0.14–1.14]	0.089
Civil servant	32	12.60	0.63	[0.23–1.77]	0.386
Student	13	5.12	0.13	[0.02–0.57]	**0.007**
**Marital status**					
Married	48	18.90	1	-	-
Divorced	138	54.33	1.09	[0.56–2.10]	0.795
Single	41	16.14	3.1	[1.25–7.7]	**0.015**
Widow/Widower	27	10.63	5.75	[1.72–19.15]	**0.004**
**Residential area**					
Urban	12	4.72	1	-	-
Rural	242	95.28	0.46	[0.12–1.76]	0.26
**Religion**					
Christian	124	48.82	1	-	-
Muslim	62	24.41	1.18	[0.63–2.21]	0.75
Endogenous	68	26.77	0.88	[0.49–1.61]	0.68
**Socio-economic well-being**					
Rich	84	33.07	1	-	-
Average	79	31.10	0.63	[0.34–1.17]	0.15
Poor	91	35.89	1.25	[0.68–2.32]	0.47
**Type of leprosy**					
PB leprosy	47	18.50	1	-	-
MB leprosy	207	81.50	7.71	[3.4–14.9]	**0.000**
**Screening mode**					
Active	88	34.65	1	-	-
Passive	166	65.35	5.94	[3.37–10.46]	**0.000**
**Family background in leprosy**					
No	177	69.69	1	-	-
Yes	77	30.31	0.66	[0.38–1.13]	0.13

### Types of leprosy, screening mode and family background

Multibacillary leprosy was predominant, accounting for 81.50% of cases (207/254). Screening was passive in 65.35% of cases (166/254). PALs had a family background of leprosy in 30.31% of cases (77/254) (Table 1).

### Understanding and perceptions

In 77.56% of cases (197/254), the PALs were not aware of the early signs and symptoms of leprosy and in 22.83% of cases (58/254), they had been afraid of being rejected by their community at the onset of the disease. For 61.42% of the respondents (156/254), leprosy was a supernatural disease with a metaphysical cause linked to a spell (Table 2).

**Table 2:** Univariate analysis of the relationship between late screening for leprosy and the knowledge, attitudes and perceptions of people affected by leprosy in Benin from 2017 to 2018.

Variables	Headcount (n = 254)	Frequency (%)	Odds Ratio	[CI _95%_ OR]	p value
**Knowledge of the early signs of leprosy**					
Knows	57	22.44	1	-	-
Do not know	197	77.56	2.95	[1.61–5.42]	**0.000**
**Knowledge of the existence of a LTC**					
Don’t know	228	89.76	1	-	-
Knows	26	10.24	0.175	[0,067–0,45]	**0.000**
**Fear of rejection**					
No	196	77.17	1	-	-
Yes	58	22.83	7.14	[3,9 – 16,50]	**0.000**
**Distance from LTC to leprosy patient’s home**					
Short (≤5km)	39	15.35	1	-	-
Long (˃5km)	215	84.65	1.28	[0.65–2.55]	0.473
**Origin of the disease**					
Natural disease	98	38.58	1	-	-
Supernatural disease related to a spell	156	61.42	10.30	[5.20–20.40]	**0.000**
**First resort**					
Self-medication	19	7.48	1	-	-
Traditional healers	149	58.66	5.21	[1.78–15.25]	**0.003**
Health agents	86	33.86	0.028	[0.007–0.11]	**0.000**

### Therapeutic pathway and time to care

Most of the PALs (149/254 or 58.66%) had consulted traditional healers at the onset of the first symptoms of the disease (Table 2).

The median time (Q1; Q3) from onset of symptoms to confirmation of leprosy diagnosis was 36 (6; 60) months. The diagnosis of leprosy was confirmed in most PALs (173/254 or 68.11%) more than 6 months after the onset of symptoms.

In 39.76% of the cases (101/254) and 68.11% of the cases (173/254) respectively, the PALs consulted more than one traditional healer and more than one health worker before the clinical diagnosis of leprosy was confirmed. (Table 3).

**Table 3:** Univariate analysis of the association between late screening and the therapeutic pathway of people affected by leprosy in Benin from 2017 to 2018.

Variables	Median (Q1, Q3)	Headcount (n = 254)	Frequency (%)	Odds Ratio	[CI _95%_ OR]	p value
**Time from first symptoms to diagnostic confirmation of leprosy (months)**	36 (6, 60)					
≤ 6		81	31.89	1	-	**-**
> 6		173	68.11	76.92	[29.41–250]	**0.000**
**Multiple visits to traditional healers before diagnostic confirmation**	2 (0, 4)					
< 2		153	60.24	1	-	**-**
≥ 2		101	39.76	5.39	[2.98–9.75]	**0.000**
**Multiple visits to health workers prior to diagnostic confirmation**	2 (1, 3)					
< 2		81	31.89	1		
≥ 2		173	68.11	4.17	[2.39–7.30]	**0.000**

### Associated factors with belated screening on leprosy patient

#### Univariate analysis

In univariate analysis, 14 variables were statistically associated with late recognition of leprosy. Among these variables were age, education, occupation, marital status, type of leprosy, mode of testing, knowledge of early signs, knowledge of LTCs, fear of rejection, perception of the cause of leprosy, first referral, time between onset of symptoms and leprosy testing, multiple visits to traditional healers and health workers. In addition, one variable had a p-value of less than 0.20, without being significantly associated, and was introduced into the logistic regression model. This was the existence or not of a family background of leprosy among the respondents (Table 1).

### Multivariate analysis

In the final model, three variables were significantly associated with late screening for leprosy in Benin. These were multiple visits to traditional healers, fear of rejection or stigma associated with the disease, and multiple visits to health workers before the clinical diagnosis of leprosy was confirmed. The risk of G2D was 5.20 times and 3.82 times higher when patients were attending more than 2 traditional healers or health workers respectively, but was 8.11 times higher when they were afraid of rejection. (Table 4). The final p-value of the Hosmer Lemshow test is 0.96, indicating the goodness of fit of the final model.

**Table 4:** Final multivariate logistic regression model showing associated factors with late screening for leprosy in Benin from 2017 to 2018.

Predictive variables	Odds Ratio	[CI _95%_ OR]	p value
**Fear of rejection**			
No	1	-	**-**
Yes	8.11	[3.3–19.94]	**0.000**
**Multiple visits to traditional healers before**			
< 2	1	-	**-**
≥ 2	5.20	[2.73–9.89]	**0.000**
**Multiple visits to health workers**			
< 2	1	-	**-**
≥ 2	3.82	[2.01–7.27]	**0.040**

### Health system factors behind the late screening for leprosy

#### Socio-demographic characteristics and level of knowledge about leprosy among health workers in the peripheral level health structures

One hundred and four (104) health workers were included in the study. The median age was 37 years with extremes ranging from 21 to 58 years. There was a male predominance of 58.65% (61/43) with a sex ratio of 1.42 (61/43). For 82.69% (18/86) of the health workers, leprosy would manifest itself early with signs of G2D (Table 5).

**Table 5:** Socio-demographic features and level of knowledge about leprosy among health workers in peripheral health structures in Benin from 2017 to 2018.

Variables	Headcount (n)	Percentage (%)
**Age (years)**		
≤ 40	67	64.42
˃ 40	37	35.58
**Sex**		
Male	61	58.65
Female	43	41.35
**Type of health workers**		
Peripheral health workers	48	46.15
LTC health workers	32	30.77
Leprosy Supervising Nurses	24	23.08
**Level of education**		
Secondary	66	63.46
University	38	36.54
**Professional status**		
Nurse	64	61.54
General practitioner	32	30.77
Medical specialist	8	7.69
**Knowledge of the pathogen**		
Knows	30	28.85
Do not know	74	71.15
**Knowledge of the early signs and symptoms of leprosy**		
Knows	18	17.31
Do not know	86	82.69
**Knowledge of the different types of leprosy according to WHO**		
Knows	32	30.77
Do not know	72	69.23
**Knowledge of the rating of disabilities in leprosy**		
Knows	40	38.46
Do not know	64	61.54
**Knowledge of the location of LTC**		
Knows	45	43.27
Do not know	59	56.73

### Operability of the NLBUCP

The following shortcomings of the NLBUCP could explain the late detection of leprosy:

- Absence of a responsible person in charge of organizing the activities of the health education, partnership and community mobilization unit;
- Retirement of trained LSIs who are proficient in leprosy, and their replacement by new agents not yet trained;
- Permanent decrease in material and financial resources allocated for leprosy control each year since the elimination threshold was reached.

### Functionality of the NLBUCP

With regard to functionality, one could note:

- The delay in the implementation of activities;
- Irregularity of formative supervision;
- Delay in the validation of cases;
- Insufficient motivation of peripheral agents.

### Factors associated with late screening for leprosy in the community

#### Description of the sample

**Fig 2:**
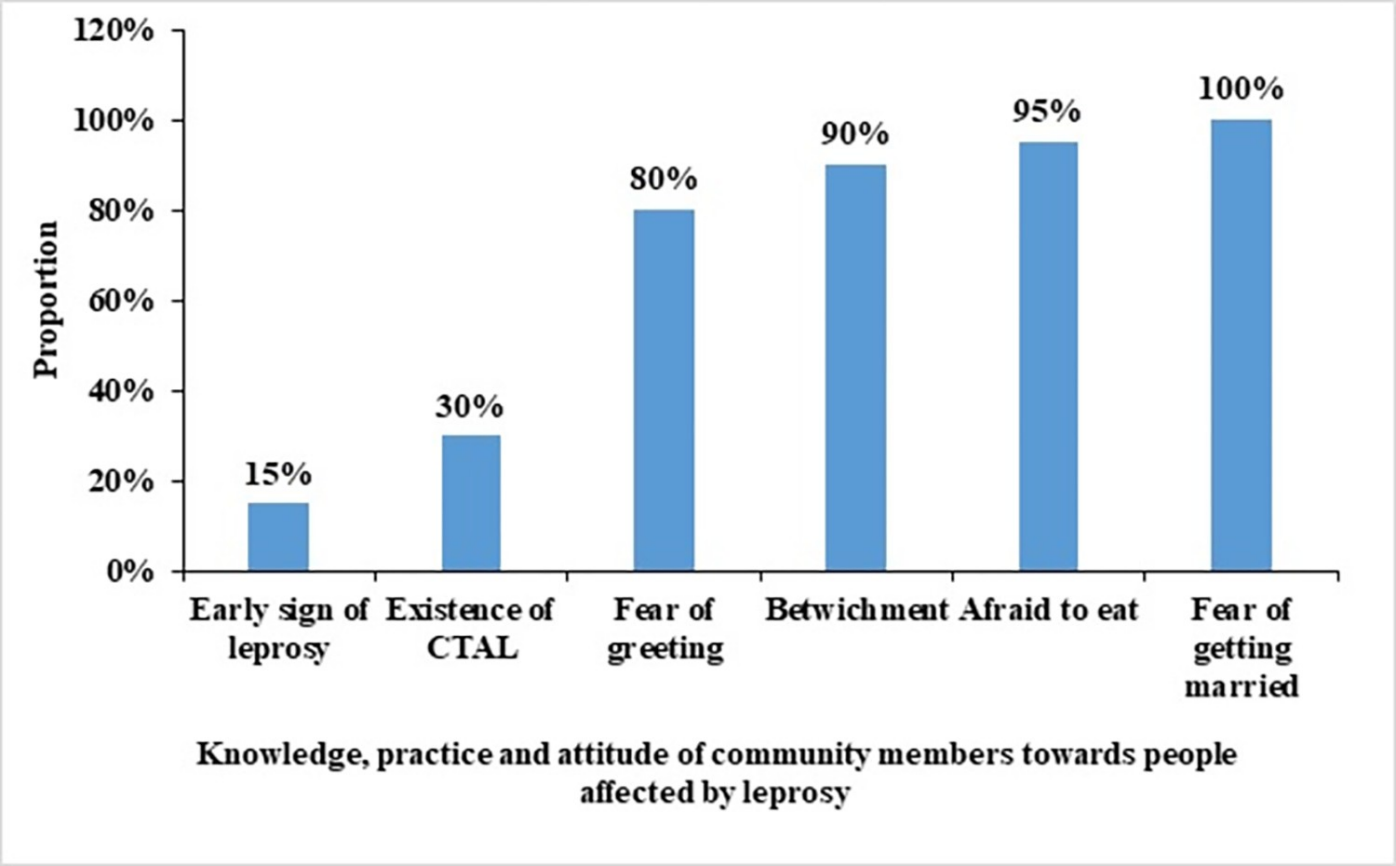
Knowledge, practice and attitude of community members towards people affected by leprosy in Benin from 2017 to 2018.

A total of 60 community members were included in the study. Their mean age was 33.7 ± 8.57. There was a 65% (39/60) female predominance with a sex ratio of 0.54 (21/39). Knowledge, attitude and practice of community members towards people affected by leprosy. All participants in the study had heard of leprosy. For 85% of the members of the community (51/60), leprosy would be manifested by G2D. Bewitchment was the main cause of leprosy in the community (54/60 or 90%). Divine punishment (4/60 or about 7%) and poor skin quality were also cited as causes of leprosy (2/60 or about 3%). In 80% of the cases (48/60), community members were afraid to greet or sit with leprosy patients and in 95% of the cases (57/60), they responded that they could not eat with leprosy patients. In 100% of the cases, the respondents categorically refused to marry leprosy patients (Fig 2).

This is justified by this statement from A. P, a young woman from the Borgou department, 26 years old: "*How can you ask this question*? *Love begins with physical attraction*. *I won’t even be able to go out with him and introduce him to my friends*, *my family; besides with all that exists as a man*, *he’s a leper and I’ll go and look for him*! *Are you guys serious*?”

**Fig 3:**
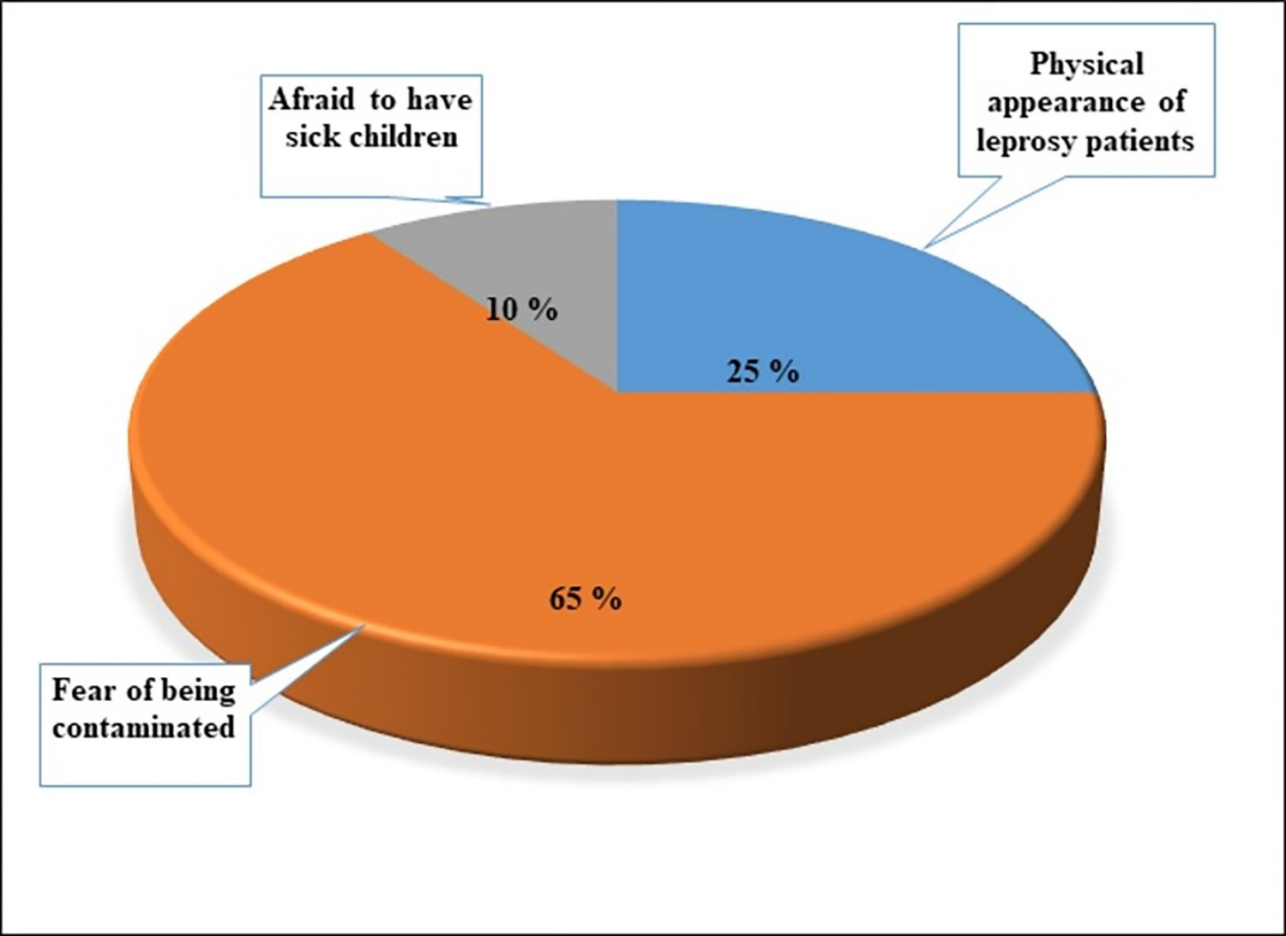
Causes of rejection of leprosy patients by community members in Benin from 2017 to 2018.

The fear of contamination (39/60 or 65%), the physical presentation of the patient (15/60 or 25%) and the fear of having sick children (6/60 or 10%) were the main reasons given by the community members to explain the rejection of people affected by leprosy (Fig 3).

## Discussion

This study allowed us to identify fear of stigma associated with leprosy (OR = 8.11; CI: 3.3–19.94; p<0.001), multiple attendance of traditional healers (OR = 5.20; CI: 2.73–9. 89; p<0.001) and attendance of multiple hospital practitioners (OR = 4.48; CI: 3.82–2.01–7.27; p<0.001) as factors associated with late screening for leprosy in Benin.

### Limitations

Limitations of this study included potential memory bias. Although steps were taken in the questionnaire to make it easier for participants to recall facts, data collection was largely dependent on the information provided by respondents. In addition, a significant proportion of participants were uneducated and the questionnaire was administered to patients from several socio-ethnic groups who did not speak the same language. This situation required the administration of an oral questionnaire and the assistance of community relays and/or community health workers to facilitate communication and its administration. Children are a vulnerable target for neglected tropical diseases and leprosy in particular. The incidence of leprosy among children is one of the direct markers of ongoing transmission of the infection in the community. Consequently, WHO’s vision, as formulated in the new roadmap for the year 2030, is to eliminate leprosy, i.e. break the chain of transmission and achieve zero new cases of pediatric leprosy by 2030. Better still, zero new cases of pediatric leprosy with grade 2 disability at screening. Hence, the absence of children in this study constitutes a selection bias that does not allow us to generalize the findings of this study to the entire socio-demographic stratum. Socio-demographic features of people affected by leprosy.

In Benin, leprosy affects relatively young subjects, making them vulnerable to the disease. Our data corroborate those reported in the literature **[14–15]**. Both sexes were affected but with a male predominance, as observed elsewhere **[10–16]**. However, our results are in contrast to those of other authors who reported a female predominance **[17–18]**. The male predominance may reflect the economic dependence of women on men **[19]**. Hansen’s disease, as well as neglected tropical diseases in general, are often correlated with low family income, low per capita income, lack of education, and poor basic health conditions **[20–22]**. Thus, the largest proportion of PALs had low education (and low socioeconomic level (83.07%) and 66.93% respectively) in accordance with previous studies **[17–23]**. This high prevalence of uneducated patients may be related to the magnitude of socio-cultural and economic inequalities between rural and urban areas in low- and middle-income countries that result in early school dropout of individuals in their communities.

In our study, our patients were mostly cultivators and moreover, they lived mostly in rural areas. Our results are in line with those of Raffé and al. who reported in Nepal in 2013 that 93% of the affected people lived in a village or rural area and that they were mostly (49%) farmers **[24]**.

According to the United Nations Development Program global report published in 2019, Benin was counted among the developing countries. It is characterized by a low (0.52) human development index (composite index that rates the average level achieved in three fundamental dimensions of human development: long and healthy life, knowledge and decent standard of living) with a multidimensional poverty rate (Percentage of the population whose deprivation score is at least 33%) estimated at 66.8% **[25]**. This situation testifies to the precarious situation of the PALs and the need for decision-makers at various levels to put in place strategies to fight poverty in order to improve the living conditions of the entire population, including leprosy patients. The implementation of these strategies could be done through the sanitation of the living environment, access to education, the promotion of small income generating activities, the granting of micro-credits to support the most vulnerable populations and all other activities that could favor the emergence of individuals living in unfavorable socio-economic conditions.

### Time from onset of symptoms to confirmation of clinical diagnosis

Our patients had a delay in seeking care (median delay (Minimum, Maximum)) of 36 (1, 192) as also observed in Brazil in 2007 (25.25 (0, 360) months) [26], as well as in Paraguay in 2003 (24 months) [27] and in China in 2009 (36 months) [28]. This delay has favored the progression of the disease to complications, resulting in a high prevalence of grade 2 disability and increased transmission of the infection in the community [28]. A major challenge for the epidemiological surveillance system is to reduce this delay in time in order to achieve the key targets of the WHO Roadmap to 2030, including the reduction of the number of new leprosy cases detected with G2D to less than one case per 1 million population by 2030 [11].

### Respondents’ level of knowledge about leprosy during the study

An overall analysis of this study reveals that leprosy patients, community members and health workers have a low level of knowledge about leprosy. It is mostly recognized by signs related to late complications of the disease. Unfortunately, it is only at this late stage that the individual recognizes him/herself as a leprosy patient and feels the need to seek consultation and treatment **[29]**. Sadly, this is a critical stage during which irreversible disabilities are established. At this stage, even regular multidrug therapy cannot restore the damaged anatomical-physiological structures, i.e. repair the disability brought on by the disease. It is at this stage of disease recognition that PALs become more vulnerable to stigmatization. Our study and others have identified the external manifestations of leprosy as one of the determinants of stigmatization, and one of the causes of rejection of leprosy patients. Physical disabilities thus further exacerbate the stigma associated with the disease, thus maintaining the cycle of stigma **[9–30]**. Stigma, cited as a cause of leprosy, also reflects the lack of knowledge about leprosy and is a testimony to the contemporary existence of misconceptions about leprosy, its signs, causes and symptoms. In other words, it reflects the lack of information and communication about leprosy. Other misbeliefs about how the disease can be contracted and its incurability have also been reported **[19–31]**. This recognition of the disease as an acquired disability reflects the lack of awareness of the early signs and symptoms of leprosy by members of the population and the need to take urgent action to strengthen the skills of health workers, educate and train the community on the signs, symptoms, causes, modes of transmission, risk factors and the availability of effective free medical treatment. It is therefore a challenge that must be faced by epidemiological surveillance actors, at the risk of jeopardizing the chances of achieving the key objectives of the WHO roadmap for the 2030 horizon.

### Associated factors with belated screening of Leprosy

Multiple visits to traditional healers, fear of stigmatization or fear of isolation, and multiple visits to hospital practitioners were the main factors associated with the occurrence of late screening for leprosy. These three factors alone accounted for 94% of the delay in leprosy testing in Benin.

### Late screening and multiple visits to traditional healers by people affected by Leprosy

For many patients in our study, leprosy is a disease caused by a spell. This reflects the psycho-social representation of leprosy within the communities and could be one of the main determinants of the patient’s therapeutic pathway. In fact, each society has its own way of thinking about the disease. In African societies, and more particularly in Benin, chronic illnesses are considered to be supernatural or metaphysical illnesses linked to a spell caused by an individual endowed with mystical power whose objective would be to harm his victim. This misconception about the causes of the disease could explain the multiple use of traditional healers in the therapeutic pathway of leprosy patients, as shown by the results of our study. This is therefore proof that, despite the modernization of medicine over the years, traditional medicine remains a non-avoidable pathway, and occupies a predominant place in the therapeutic pathway for people affected by chronic diseases such as leprosy. Due to this recourse and especially to the multiplicity of traditional healers that a leprosy patient may consult before the diagnosis is confirmed, leprosy patients have experienced long delays between the onset of clinical signs and the diagnosis of the disease by health workers. Several authors have also identified the use of a traditional healer as a factor associated with late screening for leprosy, including in Paraguay **[27]**, Ethiopia **[32]**, and Nigeria **[33]**. The multiplicity of traditional medicine practitioners has also been reported in Bangladesh, West Bengal and India **[34].**

Socio-cultural perceptions and representations shape behavior and determine therapeutic choices and practices **[35]**. In Benin, the preference for traditional healers is justified by the fact that the treatment is less restrictive, tradable and less costly, which is not the case at the health center **[36]**. This raises the whole issue of the influence of economic factors in medical recourse. The logic underlying the choice of therapeutic pathways is not only the result of the perception of the illness, but also and above all of socio-economic and health realities **[36]**.

In either case, this study shows the importance of and need for a complementary study to understand the social determinants of the decision and therapeutic pathway of leprosy patients.

Thus, the use of traditional healers would also reflect the lack of knowledge about leprosy. The role of traditional healers in the management of leprosy patients is important and should not be overlooked in the design and implementation of control interventions. It is therefore important to take into account the human being in all his or her dimensions, i.e. as a physical being, but also as a spiritual being, stemming from a community that has beliefs, rules, norms and principles.

The results of our study therefore offer avenues for research and possible innovations for collaboration between modern and traditional medicine for the holistic care of leprosy patients.

### Late screening for leprosy and fear of stigma

In our study, fear or dread of societal stigma from the community contributed to the occurrence of G2D in leprosy patients. Fear of rejection by the community had a negative impact on decisions to seek help. Leprosy patients would hide their symptoms because of this fear.

Refusal to greet or sit down, to eat or marry leprosy patients, to give them a job, fear of contamination, or having children with leprosy reflect the high level of stigma against leprosy patients in the communities. They reflect the intensity of emotional and psychological distress experienced by leprosy patients and reveal the variable areas of relational life of these patients that will be affected in society. These negative attitudes towards leprosy patients could explain the difficulties experienced by new patients in presenting themselves to the health system and the difficulties in integrating leprosy patients into their communities. The negative attitudes of community members towards leprosy patients reported in our study are comparable to those found by some authors [10,37–39]. The detrimental effects of this social stigma on the decision making of people with leprosy have also been reported by other authors. Mary Henry and al. reported in a 2016 study in Brazil that participants who suspected they had leprosy but feared isolation from the community were 10 times more apt to wait longer to see a doctor for their symptoms [40]. In Ethiopia, high rates of disability and greater delay in starting treatment were significantly associated with high levels of stigma from the community [32].

This study reveals the existence of societal stigma, for which the actors of the epidemiological surveillance system at various levels of control must develop strategies whose implementation will allow to eradicate the influence of this phenomenon within the society.

### Late screening and multiple visits by health workers

The multiplicity of hospital practitioners consulted by leprosy patients before a diagnosis of leprosy is made reveals the inadequate knowledge of health workers about the early clinical signs and symptoms of leprosy. Indeed, our study shows that there is a decrease in the number of trained health workers, resulting in a loss of expertise of health workers in diagnosing leprosy. Diagnostic errors by hospital practitioners were known to be one of the health system factors associated with late screening for leprosy. In Havana, physicians took an average of 15.6 months to reach a definitive diagnosis of leprosy in an average patient who consulted one month after the first signs appeared **[34,40,41]**. This reflects the low sensitivity of hospital practitioners to the early signs and symptoms of leprosy. Interventions should be considered to increase the level of clinical suspicion of hospital practitioners.

From the overall analysis of the results of this study, it is urgent to develop a strategic plan whose implementation will enable Benin to achieve the key objectives of the WHO roadmap for the 2030 horizon. Thus, we propose the following package of activities, to be implemented in a participatory approach, for the resolution of the identified factors.

### Communication for behavior change, with the objective of improving the knowledge, perceptions and attitudes of patients and the community on the causes, signs and early symptoms, modes of transmission and access to treatment for leprosy

We propose that the NLBUCP:

- Organize audiovisual and/or television awareness sessions on the manifestations of leprosy (signs, symptoms, complications, prevention and treatment) and collect testimonies from leprosy patients who are treated and cured. These sessions should be broadcast in several local languages to allow the entire population to access the information.
- Target and train traditional healers and faith leaders on the early signs and symptoms of leprosy and initiate a partnership for collaboration between traditional healers and health workers.
- Strengthen the training of learners in medical and paramedical sciences using innovative approaches to improve the ability to recognize and detect leprosy cases early in the community.

### Promoting the social inclusion of persons affected by leprosy, with the objective of fighting all forms of discrimination against persons affected by leprosy

This will be achieved by:

- Advocating for the need to integrate leprosy care into primary health care;
- Proposing a specific bill to ban all forms of discrimination and stigmatization against PALs
- Work to change the names of leprosy that are related to stigma.

### Increasing the NLBUCP budget

To do so, it will be necessary to:

- Make a plea to draw the attention of political and administrative authorities and technical and financial partners to the need to increase the budget allocated to the NLBUCP
- Diversify the program’s funding sources.

### Promotion of active case and contact tracing

This will involve organizing:

- Periodic mobile clinics in endemic regions for integrated screening of neglected tropical skin diseases;
- Survey of MB cases and contacts of Leprosy patients;

### Improving the quality of screening and management of people affected by leprosy

- To provide training to health workers in dermato-leprology to serve the community in leprosy care centers.
- Strengthen the training of medical and paramedical learners using innovative approaches to improve the ability to recognize and detect leprosy cases early in the community
- Conduct periodic capacity building of peripheral level health workers and LTCs
- Equip the LCTs with mobile and medical technology equipment for diagnostic confirmation and management of leprosy patients.
- To set up a motivation scheme for the LSNs and LTC agents in this particular context of decreasing resources, it will be necessary to orient and organize all these activities in the endemic areas of the disease.

## Conclusion

In Benin, the fight against leprosy remains a health challenge. Difficulties are linked to the limitations of financial, human and material resources. Our research findings are fundamental pillars which could help the actors of the leprosy epidemiological surveillance system in developing new innovative strategies and implementing activities to achieve and accelerate their progress towards the key objectives of the new roadmap developed by WHO for 2030. These activities should take into account the reduction of stigmatization of PALs, sensitization of communities on the early signs and symptoms of leprosy and capacity building of health workers so that by 2030, Benin will be counted among the countries that have achieved "zero leprosy", i.e. zero infection, zero disease, zero disability, zero stigma and zero discrimination [42]. To achieve this, the commitment of all stakeholders is essential to meet these challenges.

## Compliance with guidelines and regulations

We confirm that all methods used in this observational study complies with all research guidelines and regulations. All the experiment protocol for involving humans in this manuscript are in accordance to guidelines of national/international/institutional and the Declaration of Helsinki.
